# Hard Physical Work Intensifies the Occupational Consequence of Physician-Diagnosed Back Disorder: Prospective Cohort Study with Register Follow-Up among 10,000 Workers

**DOI:** 10.1155/2017/1037051

**Published:** 2017-01-31

**Authors:** Emil Sundstrup, Lars Louis Andersen

**Affiliations:** ^1^National Research Centre for the Working Environment, Copenhagen, Denmark; ^2^Physical Activity and Human Performance Group, SMI, Department of Health Science and Technology, Aalborg University, Aalborg, Denmark

## Abstract

While musculoskeletal pain is common in the population, less is known about its labor market consequences in relation to physical activity at work. This study investigates whether hard physical work aggravates the consequences of back disorder. Using Cox regression analyses, we estimated the joint association of physical activity at work and physician-diagnosed back disorder in 2010 with the risk of register-based long-term sickness absence (LTSA) of at least 6 consecutive weeks during 2011-2012 among 9,544 employees from the general working population (Danish Work Environment Cohort Study). Control variables were age, gender, psychosocial work environment, smoking, leisure physical activity, BMI, depression, and mental health. At baseline, 19.4% experienced high low-back pain intensity (≥5, 0–9 scale) and 15.2% had diagnosed back disorder. While high pain intensity was a general predictor for LTSA, physician-diagnosed back disorder was a stronger predictor among those with hard physical work (HR 2.23; 95% CI 1.68–2.96) compared with light work (HR 1.40; 95% CI 1.09–1.80). Similarly, physician-diagnosed back disorder with simultaneous high pain intensity predicted LTSA to a greater extent among those with hard physical work. In conclusion, the occupational consequence of physician-diagnosed back disorder on LTSA is greater among employees with hard physical work.

## 1. Introduction

Back disorder and musculoskeletal disorders in general is a huge public health problem, limiting productivity at work and imposing a substantial socioeconomic burden on the society. Most individuals will experience one or more episodes of low-back pain (LBP) during their lifetime, and for approximately 10%, LBP persists over time and becomes chronic, leading to work disability, sickness absence, or loss of employment [1–3]. The Global Burden of Disease Study shows that back pain is among the top five leading causes of disability worldwide [4]. With expectations of higher retirement age in most countries, the burden from back disorder is likely to increase in future years [1, 5]. Thus, increased knowledge to better understand and manage back disorder may provide better opportunities for preventing premature exit from the labor market.

Back disorder has a multifactorial etiology consisting of a complex interaction between individual factors and physical and psychosocial work environmental factors [6–10]. In the physical work environment, factors such as hard physical work involving heavy lifting, bending and twisting of the back, and awkward postures have been identified as risk factors for the development of back disorders, such as herniated disc and chronic LBP [11–14]. Several risk factors for back disorder are therefore present during hard physical work, which is also mirrored in the high prevalence of back disorder among this group of workers. However, less is known about the consequences of existing back disorder on work related outcomes in relation to the degree of physical activity at work. We have previously found that workers with hard physical work are more likely to use medication on a regular basis for musculoskeletal disorders, including low-back pain, which suggests that the consequences of back pain are higher among individuals with hard physical work [15]. Thus, the level of physical activity at work may have implications for how back disorder is managed and for the consequence of the disease in regard to labor market attachment.

Back pain symptoms are rarely attributed to a specific pathology. Thus, in 9 out of 10 cases identifying the pathology is not possible and the majority of cases are therefore considered as nonspecific back pain [3, 16]. However, factors such as lumbar disc degeneration seen with clinical imaging have been associated with low-back pain [16, 17]. Seeking care because of low-back pain could be an indication of the seriousness of the condition, that is, how much it interferes with activities of daily living such as work and social activities. In this regard, Mortimer and Ahlberg (2003) showed that the most decisive factors for seeking care due to low-back pain were high disability and pain intensity [18]. However, the impact of back disorder could be different for workers engaged in hard physical work compared with light physical work. Having back disorder in combination with a physically demanding job may lead to greater difficulties in meeting physical work demands and thereby challenge the capacity to participate in gainful employment [19]. To better prevent premature exit from the labor market among different occupational groups, knowledge about the influence of physical activity at work on the consequences of back disorder is needed.

This study aims to determine the joint association of physician-diagnosed back disorder, back pain, and physical activity at work with the risk of LTSA. We hypothesized that the consequence of physician-diagnosed back disorder and back pain on the risk of LTSA is higher among employees engaged in hard physical work compared with workers with light physical work.

## 2. Materials and Methods

### 2.1. Study Design

By merging data from the Danish Work Environment Cohort Study (DWECS) with the national register of social transfer payment (DREAM), this study estimates the joint association of physician-diagnosed back disorder, back pain, and physical activity at work with the risk of LTSA.

### 2.2. Study Population

Questionnaire data on health and work environmental factors from the 2010 round of the DWECS [20] was used for the present study. In 2010, 10,605 workers (~53%) replied to the questionnaire survey [21]. For the present study, only currently employed wage earners that were free from LTSA in 2009 and 2010 were included (*n* = 9,544). Since not all participants filled in all the survey questions, the exact number of workers included in each analysis varies. Characteristics of the study population at baseline can be seen in Table 1.

### 2.3. Ethical Approval

The study was notified and registered by the Danish Data Protection Agency (journal number: 2007-54-0059) and all data were deidentified and analyzed anonymously. According to Danish law, register-based and questionnaire-based studies do not need informed consent or approval by ethical and scientific committees [22, 23].

### 2.4. Predictor Variables

The following describes the predictor variables included in the statistical analyses.

#### 2.4.1. Physician-Diagnosed Back Disorder

Physician-diagnosed back disorder was identified by the following question, “Have you ever been informed by a physician that you have or have had one or more of the following conditions?” with the response options being “Yes” and “No, never” to back disorder [24].

#### 2.4.2. Back Pain Intensity

Back pain intensity was assessed as average low-back pain during the last 3 months on a scale of 0–9, where 0 is no pain and 9 is worst pain. Participants replied to this question regardless of whether they had a physician-diagnosed back disorder. The pain question was phrased as “trouble (pain or discomfort).” [25] For further analyses, back pain was trichotomized into “No pain” (pain intensity 0–2), “Moderate pain” (pain intensity 3-4), and “High pain” (pain intensity ≥5).

#### 2.4.3. Physical Activity at Work

Physical activity at work was assessed by a question from DWECS with the following response categories: (1) “Mainly sedentary work,” (2) “Mainly standing or walking work that is not strenuous,” (3) “Standing or walking work with lifting/carrying tasks,” (4) and “Heavy or fast strenuous work.” For the analyses in the present study, response option 1 and 2 were collapsed and defined as “Light physical work,” and options 3 and 4 as “Hard physical work.”

### 2.5. Outcome Variable

The data on long-term sickness absence used in the present study was derived from The Danish Register for Evaluation and Marginalization (DREAM), which contains information on all social transfer payments (including sickness absence compensation, unemployment benefits, and early retirement) for all Danish residents on a weekly basis [26, 27]. Questionnaire survey data from DWECS were prospectively linked to DREAM by the unique personal identification number given to all Danish citizens at birth. In Denmark, sickness absence benefit is paid to individuals who are unable to work due to illness or who have been injured and before the period of sickness absence had some connection to the labor market. In addition, the employer has the right to ask for proof of sick leave by a medical certificate. Furthermore, employers can apply for government compensation of sickness absence costs after 30 days of sickness absence, whereas we defined LTSA as sickness absence as more than 30 calendar days, corresponding to ≥6 consecutive weeks in DREAM during the 2-year follow-up period (2011-2012).

### 2.6. Control Variables

Control variables for the analyses in the present study included age, gender, psychosocial work environment (influence at work, emotional demands, support from colleagues, and support from leaders), smoking status (“No, never,” “Ex-smoker,” and “Yes”), physical activity during leisure (low, moderate, or high), body mass index (BMI), depression (have you ever been informed by a physician that you have or have had depression?), and mental health (from the SF-6 questionnaire) [15, 28].

### 2.7. Statistics

We used Cox proportional hazard models for modelling the risk of LTSA during the 2-year follow-up period. The data on LTSA corresponds to survival times and participants were censored in case of retirement, disability pension, immigration, or death. The Danish Work Environment Cohort was followed for 2 years after the baseline year (i.e., 2011 and 2012), and when participants had an onset of LTSA within this period, the survival times were noncensored and referred to as event times. Individuals with an episode of LTSA during the two years prior to baseline were excluded from the analysis. The estimation method was maximum likelihood and the results are reported as hazard ratios (HR) with 95% confidence intervals (CI).

The analysis presented in Table 2 shows the prospective associations between different combinations of back pain intensity and physical activity at work for the risk of LTSA. In this analysis, model 1 was adjusted for age and gender. Model 2 was adjusted for the same as model 1 but additionally adjusted for psychosocial work environment. Model 3 was adjusted for the same as model 2 but additionally adjusted for lifestyle (smoking, leisure physical activity, and BMI). Model 4 was adjusted for the same as model 3 but additionally adjusted for depression and mental health.

The analysis presented in Table 3 shows the prospective associations between different combinations of physician-diagnosed back disorder and physical activity at work for the risk of LTSA. The 4 models in the analysis were adjusted according to the models mentioned above (Table 2).

The analysis presented in Table 4 shows the prospective associations between different combinations of back pain, physician-diagnosed back disorder, and physical activity at work for the risk of LTSA. For this analysis, only participants with no back pain and high back pain were included to limit the number of comparisons. The 4 models in the analysis were adjusted according to the models mentioned above (Table 2).

## 3. Results

Of the 9.544 participants, 28.2% had hard physical work. Further, 15.2% had a physician-diagnosed back disorder, and 19.4% experienced high back pain (intensity ≥5). Descriptive statistics for the study population is shown in Table 1.

Table 2 shows the prospective associations between back pain intensity and physical activity at work for the risk of LTSA. In the fully adjusted model 4, the risk for LTSA increased with increasing pain intensity for both light and hard physical work.

Table 3 shows the prospective associations between physician-diagnosed back disorder and physical activity at work for the risk of LTSA. Having a diagnosed back disorder increased the risk for LTSA to a greater extent for employees engaged in hard physical work (HR: 2.23; CI95: 1.68–2.96) compared with workers engaged in light work (HR: 1.40; CI95: 1.09–1.80).

Table 4 shows the prospective associations between back pain, physician-diagnosed back disorder, and physical activity at work for the risk of LTSA. In the fully adjusted model, having both high pain (intensity ≥5) and diagnosed back disorder increased the risk estimate for LTSA to a greater extent for employees engaged in hard physical work compared with those with light work; HR: 2.80 (CI95: 1.93–4.06) versus 1.81 (1.27–2.56).

In all the analyses, performing hard physical work increased the risk estimates for LTSA compared with light work (Tables 2, 3, and 4).

## 4. Discussion

While pain intensity was a general predictor for LTSA, physician-diagnosed back disorder was a stronger predictor among those with hard physical work compared with light physical work. Importantly, the combination of back disorder and high low-back pain intensity predicted LTSA to a greater extent among employees with hard physical work. Overall, the occupational consequence of physician-diagnosed back disorder on LTSA seems to be greater among employees with hard physical work.

Before presenting and discussing the results, we will address some limitations and strengths to the study. A limitation of the study is that, due to Danish law, the DREAM register contains no information regarding the cause of sickness absence. Thus, even though physician-diagnosed back disorder was associated with increased risk of LTSA, we have no information about the cause of each case of sickness absence. Another limitation is that diagnosed back disorder was obtained by self-report rather than by the Danish hospitalization register. However, the questionnaire specifically stated that the back disorder should have been diagnosed by a physician, which likely reduces reporting bias. In addition, the Danish hospitalization register only includes individuals who have been hospitalized due to back disorder, that is, relatively serious cases, which may underestimate the total number of individuals with a back disorder.

The strength of the study is the step-wise addition of control variables thought to be potential confounders; that is, first we adjusted for psychosocial factors, then lifestyle, and finally depression and mental health. Because strong associations previously have been observed between psychosocial work factors such as low influence at work, increased risk of LTSA [29–31], and back disorder [32, 33], we included psychosocial work environment as a control variable in the analyses. However, this did not change the risk estimates in any of the analyses (model 2 in Tables 2, 3, and 4), suggesting that psychosocial work environment might not be central for determining LTSA among workers with back disorder and/or back pain. Adjusting for lifestyle factors (smoking, leisure physical activity, and BMI) reduced the risk estimates for LTSA in all the analyses (model 3 in Tables 2, 3, and 4). Smoking, leisure physical activity, and BMI are considered modifiable factors associated with back pain and also seem to be important confounders in the present study [34]. Depression and mental health were added to the analysis in the final model, which led to a reduction of the risk estimates for LTSA (model 4 in Tables 2, 3, and 4). This is in agreement with previous studies showing that comorbid depression amplifies the negative impacts of musculoskeletal disorders and that back problems and comorbid depression lead to high negative impact on employment and work participation [35, 36]. Altogether, our results highlight the relevance of adjusting for lifestyle, comorbid depression, and mental health when investigating the association between back disorder and sickness absence.

Another strength of the study is the use of information on sickness absence from the DREAM register. The DREAM register has a high validity since employers have an economic incentive to report sickness absence as employers can apply for compensation of sickness absence costs after 30 days of sickness absence. This inherently eliminates any reporting or recall bias. Another strength is the inclusion of currently employed wage earners that were free from LTSA over the preceding two years prior to data collection (i.e., baseline). This inherently eliminates cross-sectional comparisons and any bias that would arise specifically if those on LTSA reported their pain intensity differently from those who were not on sick leave. Finally, the use of a large representative sample of the general working population in Denmark increases the generalizability of the study.

With these limitations and strengths in mind, we observed that physician-diagnosed back disorder was a stronger predictor for LTSA among those with hard physical work compared with light work. While previous studies have identified several risk factors in the physical work environment for the development of back disorder [11–14], our study shows that the level of physical activity at work has important implications for the occupational consequence of back disorder in regard to future sickness absence. Having a back disorder in combination with a physically demanding job may lead to difficulties in meeting physical work demands and thereby reduces work ability and increases the risk of sickness absence. In addition, hard physical work may aggravate existing pain making it even harder to meet the physical requirements of the job.

In the final set of analyses we combined physician-diagnosed back disorder and pain intensity (Table 4). In that analysis the combination of having diagnosed back disorder and high pain intensity predicted LTSA to a greater extent among those with hard physical work. Overall taken, to reduce future LTSA among workers with physician-diagnosed back disorder, initiatives to reduce the impact of the disease should especially target employees engaged in hard physical work. Hence, clinicians should recommend appropriate job modifications for their back pain patients with heavy physical work. Thus adjusting the demands at work to fit the individual worker could be a strategy to reduce sickness absence among work with a back disorder. Reducing exposure to physical demanding work could be accomplished by organizing the work another way, for example, by the use of technical aids when appropriate (such as lifting devices) and/or by incorporating microbreaks or job rotation to less physically demanding job tasks. Workplace policies should therefore ensure that workers with back disorder have the immediate opportunity to reduce the physical workload. Future studies should investigate which initiatives are most effective at reducing physical workload and/or increasing the capacity of the worker to secure work ability among employees with back disorder and hard physical work.

Pain intensity, when not considering a specific diagnosis, was prospectively associated in a progressive fashion with LTSA both among workers with light and hard physical work. This is in agreement with a previous study identifying severe low-back pain as a general risk factor for LTSA among both blue- and white-collar workers [37]. We have previously shown that the odds for using medication on a regular basis for musculoskeletal pain, including low-back pain, are higher among employees with a high degree of physical activity at work, even when controlling for pain intensity [15]. Thus, it seems likely that the level of physical activity at work could have implications for how musculoskeletal pain is managed by the individual worker and for the occupational consequence of the pain. However, in the present study the prospective association between low-back pain intensity and LTSA was not significantly stronger among those with hard physical work (Table 2). Thus, pain intensity by itself can be considered a general predictor of LTSA.

There were also some secondary findings of the present study. Workers with hard physical work had a higher risk of LTSA than workers with light physical work. This is congruent with previous studies showing that exposure to high physical work demands such as heavy lifting or carrying, bending or twisting of the back, and monotonous movements are risk factors for long-term sickness absence [38–42]. In addition, Andersen et al. [42] reported that a higher number of combined exposures to different physical workloads were associated with progressively higher risk for LTSA. The present study elaborates on these previous findings by showing that hard physical work is also a risk factor for LTSA for workers with no pain. Thus, the risk for LTSA was 42% higher among those with hard physical work compared with light physical work (Table 2).

## 5. Conclusion

The occupational consequence of physician-diagnosed back disorder was greater among employees with hard physical work. Thus, diagnosed back disorder was a stronger predictor for LTSA among those with hard physical work compared with light work. Importantly, the combination of back disorder and high low-back pain intensity predicted LTSA to a greater extent among employees with hard physical work. Future interventions aiming at reducing the occupational impact of back disorder should especially target employees engaged in hard physical work.

## Figures and Tables

**Table 1:** Description of the study population at baseline.

	*N*	Mean (SD)	%
Age, years	9544	43.2 (11.8)	
Gender			
Men	4479		46.9
Women	5065		53.1
Physical activity at work			
Light	6655		71.8
Hard	2611		28.2
Physician-diagnosed back disorder			
No	7915		84.9
Yes	1413		15.2
Back pain intensity			
0–2	5605		59.9
3-4	1933		20.7
5–9	1816		19.4
Psychosocial work factors (0–100)			
Emotional demands	9295	44.2 (24.9)	
Influence at work	9238	67.8 (23.9)	
Support from colleagues	8657	73.2 (21.4)	
Support from leader	8871	69.9 (25.6)	
Smoking			
No, never	4557		49.0
Ex-smoker	2627		28.3
Yes	2113		22.7
Physical activity during leisure			
Low	1230		13.2
Moderate	6259		67.4
High	1800		19.4
BMI (kg·m^−2^)	9233	25.3 (4.3)	
Depression			
No	8320		89.1
Yes	1014		10.9
Mental health (0–100)	9326	81.1 (14.5)	
Long-term sickness absence during follow-up			
Yes	703		7.4
No	8841		92.6

**Table 2:** The prospective associations between back pain intensity, physical activity at work, and risk of LTSA.

	*N*	%	Model 1	Model 2	Model 3	Model 4
			HR (95% CI)	HR (95% CI)	HR (95% CI)	HR (95% CI)
Pain 0–2, light work	4272	46.9	1.00	1.00	1.00	1.00
Pain 3-4, light work	1244	13.7	1.33 (1.04–1.70)	1.36 (1.05–1.76)	1.33 (1.03–1.72)	1.30 (1.00–1.68)
Pain 5–9, light work	1035	11.4	2.15 (1.72–2.68)	2.06 (1.63–2.61)	1.90 (1.49–2.42)	1.74 (1.36–2.22)
Pain 0–2, hard work	1210	13.3	1.38 (1.07–1.78)	1.46 (1.12–1.91)	1.40 (1.07–1.83)	1.42 (1.08–1.86)
Pain 3-4, hard work	630	6.9	1.72 (1.28–2.30)	1.71 (1.25–2.33)	1.61 (1.18–2.20)	1.60 (1.17–2.18)
Pain 5–9, hard work	716	7.9	2.74 (2.16–3.48)	2.71 (2.09–3.50)	2.44 (1.87–3.17)	2.20 (1.68–2.87)

Model 1: adjusted for age and gender.

Model 2: model 1 + psychosocial work environment (influence at work, emotional demands, support from colleagues, and support from leader).

Model 3: model 2 + lifestyle (smoking, leisure physical activity, and BMI).

Model 4: model 3 + depression and mental health.

**Table 3:** The prospective associations between physician-diagnosed back disorder, physical activity at work, and risk of LTSA.

	*N*	%	Model 1	Model 2	Model 3	Model 4
			HR (95% CI)	HR (95% CI)	HR (95% CI)	HR (95% CI)
No back disorder, light work	5609	61.8	1.00	1.00	1.00	1.00
Back disorder, light work	928	10.2	1.57 (1.24–1.97)	1.55 (1.21–1.97)	1.48 (1.16–1.90)	1.40 (1.09–1.80)
No back disorder, hard work	2097	23.1	1.44 (1.20–1.73)	1.42 (1.17–1.73)	1.34 (1.10–1.64)	1.32 (1.08–1.62)
Back disorder, hard work	445	4.9	2.37 (1.81–3.11)	2.48 (1.87–3.29)	2.36 (1.78–3.13)	2.23 (1.68–2.96)

Model 1: adjusted for age and gender.

Model 2: model 1 + psychosocial work environment (influence at work, emotional demands, support from colleagues, and support from leader).

Model 3: model 2 + lifestyle (smoking, leisure physical activity, and BMI).

Model 4: model 3 + depression and mental health.

**Table 4:** The prospective associations between back pain intensity, physician-diagnosed back disorder, physical activity at work, and risk of LTSA. “No pain” refers to back pain intensity of 0–2 (0–9 scale) and “High pain” as back pain intensity of ≥5.

	*N*	%	Model 1	Model 2	Model 3	Model 4
			HR (95% CI)	HR (95% CI)	HR (95% CI)	HR (95% CI)
No pain, no back disorder, light work	3990	55.4	1.00	1.00	1.00	1.00
No pain, back disorder, light work	1109	15.4	1.30 (0.81–2.09)	1.35 (0.82–2.23)	1.37 (0.83–2.26)	1.33 (0.81–2.19)
High pain, no back disorder, light work	268	3.7	2.18 (1.66–2.85)	2.10 (1.57–2.80)	1.98 (1.48–2.65)	1.85 (1.38–2.48)
High pain, back disorder, light work	88	1.2	2.29 (1.67–3.13)	2.21 (1.58–3.08)	1.97 (1.39–2.78)	1.81 (1.27–2.56)
No pain, no back disorder, hard work	635	8.8	1.34 (1.02–1.75)	1.38 (1.04–1.84)	1.35 (1.02–1.81)	1.36 (1.02–1.81)
No pain, back disorder, hard work	471	6.5	1.96 (1.00–3.82)	2.02 (0.99–4.10)	2.09 (1.03–4.26)	2.11 (1.04–4.30)
High pain, no back disorder, hard work	396	5.5	2.58 (1.92–3.46)	2.43 (1.77–3.33)	2.16 (1.56–3.00)	1.96 (1.41–2.74)
High pain, back disorder, hard work	240	3.3	3.29 (2.33–4.64)	3.32 (2.31–4.77)	3.05 (2.12–4.41)	2.80 (1.93–4.06)

Model 1: Adjusted for age and gender.

Model 2: model 1 + psychosocial work environment (influence at work, emotional demands, support from colleagues, and support from leader).

Model 3: model 2 + lifestyle (smoking, leisure physical activity, and BMI).

Model 4: model 3 + depression and mental health.
